# Comparative study of antibiotic adsorption capacity of three brands of blood culture bottles

**DOI:** 10.3389/fcimb.2025.1571264

**Published:** 2025-10-08

**Authors:** Siqi Yuan, Jinxue Yang, Nan Deng, Jiayi Peng, Ling Ma, Shuaixian Du

**Affiliations:** Department of Clinical Laboratory, Union Hospital, Tongji Medical College, Huazhong University of Science and Technology, Wuhan, China

**Keywords:** bloodstream infection, blood culture, blood culture system, antibiotic adsorption, time to detection

## Abstract

**Purpose:**

To compare the adsorption capacity of BacT/ALERT FA Plus aerobic, FN Plus anaerobic bottles, BD BACTEC Aerobic/F Plus, resin-free Lytic/10 Anaerobic/F bottles and VersaTREK Aerobic, Anaerobic bottles for antibiotics.

**Methods:**

The clinically commonly used vancomycin, cefoperazone/sulbactam, imipenem and penicillin were investigated in the study. These antibiotics were mixed with standard strains and sterile horse blood at certain concentrations and inoculated into aerobic and anaerobic bottles of the three blood culture systems, then placed in the automated blood culture instrument. The positive detection rate and time to detection (TTD) of these three blood culture systems were recorded within five days in order to compare their detection and adsorption of antibiotics. Finally, one of the most detectable blood culture bottles were used on patients in the comprehensive intensive care unit (ICU) for one month and analyzed retrospectively in comparison with previous data.

**Results:**

The detection rates of the aerobic bottles were 25/35 (71.4%) for BacT/Alert FA Plus and BACTEC Aerobic/F Plus bottles, 12/35 (34.3%) for VersaTREK Aerobic bottles. The detection rates of the anaerobic bottles were 20/35 (57.1%) for BacT/Alert FN Plus bottles, 1/35 (2.9%) for VersaTREK Anaerobic bottles, and no positive bottles were detected in BD BACTEC Lytic/10 Anaerobic bottles. The TTD for the BacT/Alert FA Plus bottle (24.4 hours) was 5.1 hours shorter than the BD BACTEC Aerobic/F Plus bottle (29.5 hours) and 34.1 hours shorter than the VersaTREK Aerobic bottle (58.5 hours). In the ICU, the detection rates were 12.5% and 9.1% for BacT/ALERT medium and VersaTREK medium, respectively. For gram-negative bacteria, the TTD for BacT/ALERT medium and VersaTREK medium were 19.02 hours and 18.29 hours, respectively. For gram-positive bacteria, the TTD for BacT/ALERT medium and VersaTREK medium were 22.25 and 35.87 hours, respectively.

**Conclusion:**

The BacT/ALERT system has relative advantages in the detection rate, TTD, and antibiotic adsorption capacity, and can be used selectively in patients who have received antibiotic therapy. The use of BacT/ALERT medium resulted in a relative increase in detection rates, as well as in the type and number of strains detected compared to the VersaTREK medium previously used in the comprehensive ICU.

## 1 Introduction

Bloodstream infections (BSIs) is a major public health problem worldwide and produce a high mortality rate. Rapid and accurate diagnosis of pathogens allows patients to receive effective antimicrobial therapy immediately and is effective in reducing the mortality rate of BSIs (Lamy et al., 2020). Although new microbiological identification techniques are under constant development, blood cultures remain the gold standard for the diagnosis of BSIs, and should be collected from patients with suspected BSIs prior to the administration of antimicrobial therapy. However, worldwide surveys have shown low compliance rates for collecting blood cultures from patients with potentially serious infections prior to antibiotic administration. For patients in the ICU, physicians often use antibiotics empirically before blood culture results are available (Ferrer et al., 2008; Phua et al., 2011; Rhodes et al., 2015). Notably, culture positivity has been shown to be reduced by 20% in blood cultures obtained during antibiotic therapy (Scheer et al., 2019). Commercial blood culture bottles are normally recommended for incubation in instruments for 5 days, resulting in extended detection times. This is not conducive to timely optimization of patient treatment regimens.

Some commercial blood culture media incorporate antibiotic-absorbent substances, such as resins or activated carbon, to minimize the inhibition of microbial growth by antibiotics in blood cultures. Our microbiology laboratory currently has three commercial automated blood culture systems, including BacT/ALERT, BD BACTEC and VersaTREK blood culture system. BacT/ALERT and BD BACTEC aerobic bottles culture media contain resins that adsorb free antibiotics from the blood, and VersaTREK and BD BACTEC anaerobic bottles culture media contain hemolysins that dilute antibiotics; these additives have been proven to improve the positive detection rate.

In this study, we compared the antibiotic adsorption capacity of three blood culture systems, BacT/ALERT, BD BACTEC, and VersaTREK, and evaluated the detection rate and time to detection (TTD) of various antibiotic-bacterial combinations in simulated blood cultures. The detection rate of positive blood cultures and the types of bacteria detected from June 2022 to June 2023 were also compared in a comprehensive cohort of ICU patients.

## 2 Materials and methods

### 2.1 Blood culture media and instruments

Resin-containing BacT/ALERT FA Plus (FA Plus) aerobic bottles, FN Plus (FN Plus) anaerobic bottles were applied to the automated BacT/ALERT 3D blood culture system (Bio Mérieux, France). Resin-containing BD BACTEC Aerobic/F Plus (BD Aerobic Plus) bottles and resin-free Lytic/10 Anaerobic/F (BD Lytic/10 Anaerobic) bottles were attached to the automated BD BACTEC FX blood culture system (Becton, Dickinson and Company, the United States). VersaTREK Aerobic bottles and Anaerobic bottles were attached to the automated Thermo Scientific VersaTREK System (Thermo Fisher Scientific, the United States). The three blood culture instruments in this study were started to use on May 10, 2015. We conduct performance verification on the blood culture instruments once a year. The performance verification was conducted on July 26, 2022 and July 26, 2023 for each blood culture instrument.

### 2.2 Strains

Microbial species were selected according to the detection rate of positive blood cultures in our laboratory, with the following ATCC microbial strains (obtained from American Type Culture Collection) being used: *Escherichia coli* ATCC 25922; *Pseudomonas aeruginosa* ATCC 27853; methicillin-susceptible *Staphylococcus aureus* ATCC 29213; and *Streptococcus pneumoniae* ATCC 49619. The standard strains were inoculated onto Columbia blood agar plates twice, cultured at 37°C for 18–24 h. Individual colonies were prepared in saline to make a suspension of 0.5 McFarland Standard turbidity and sequentially diluted multiplicatively to prepare a final concentration of 100 colony-forming unit (CFU)/mL. We prepared the 100 CFU/mL concentration of bacterial suspension by multiple dilution method. First, pick a single colony and prepare a bacterial suspension with a turbidity of 0.5 McFarland Standard turbidity using physiological saline. At this point, the bacterial concentration is approximately 10^8^ CFU/mL. Then take three sterile sample tubes, numbered sequentially as 1, 2, and 3, and add 9.9 ml, 9.9 ml, and 11.88 ml of physiological saline respectively. Transfer 100 μL of the prepared bacterial suspension at 0.5 McFarland standard into Tube 1. After mixing, the bacterial suspension in Tube 1 contained 10^6^ CFU/mL. Next, transfer 100 μL of the bacterial suspension from Tube 1 into Tube 2 to obtain a suspension with a concentration of 10^4^ CFU/mL. Finally, add 120 μL of the bacterial suspension from Tube 2 to Tube 3 to obtain a suspension with a concentration of 100 CFU/mL. For each set of experiments, we prepared 12 ml of bacterial suspension for later use. An aliquot of 0.3 mL of the diluted bacterial suspension was taken and inoculated onto Columbia blood agar plates and incubated overnight at 37°C to verify CFUs.

### 2.3 Antibiotics

In this study, four of the most commonly used antimicrobials were selected to treat simulated BSIs. We purchase antibiotic powders including imipenem (MB1457-S), cefoperazone (MB1805-1), sulbactam (MB5725-S), penicillin (MB2047-S) and vancomycin (MB1260-S) from Meilunbio, China. According to the instructions, select an appropriate solvent. Calculate the antibiotic concentration for the experiment based on the peak serum concentration of antibiotics from THE SANFORD GUIDE TO ANTIMICROBIAL THERAPY, and prepare the antibiotic solution for the experiment.

### 2.4 Blood culture preparation

In simulated blood culture experiments, 10 mL of sterile horse blood (obtained from Oxoid Limited, Britain), 0.3 mL of prepared bacterial suspension and 0.3 mL of antibiotic solution were added to each blood culture bottle. For each bacteria-antibiotic combination, five replicates were performed for different types of blood culture bottles. In addition, a positive control without antibiotics was set up for each combination, with three replicates of each control group. Subsequently, the culture bottles were incubated in the blood culture instrument, and the positive alarms and detection times of the different bottles recorded for five days. A negative result was the blood cultures not detecting a signal of bacterial growth within five days, and a positive result was the detection of a signal of bacterial growth within five days and an alarm. All positive blood cultures were subcultured onto Columbia blood agar plates for overnight incubation, and the isolated bacteria were identified using matrix-assisted laser desorption ionization time-of-flight mass spectrometry (MALDI-TOF).

### 2.5 Data analysis and statistics

Statistical analysis was performed using GraphPad Prism 6. Fisher’s exact test was used to compare the detection rates of the three culture systems between aerobic and anaerobic media. The Mann-Whitney test analysis was used to compare the TTD. *P*-value<0.05 was considered to be a statistically significant finding.

## 3 Results

We evaluated 210 BacT/ALERT (FA Plus and FN Plus), BD BACTEC (Aerobic/F Plus and Lytic/10 Anaerobic/F), and VersaTREK (Aerobic and Anaerobic) blood culture bottles to assess the adsorbed antibiotic functions (**Table 1**). The detection rate of BacT/ALERT system was 45/70 (64.3%), for the BD BACTEC system 25/70 (35.7%) and for the VersaTREK system 13/70 (18.6%). In the positive control without antibiotics, all bacteria were detected. For aerobic bottles, both the BacT/ALER system and the BACTEC system produced a detection rate of 25/35 (71.4%), while the VersaTREK system had a detection rate of 12/35 (34.3%) (*P*=0.047). In anaerobic bottles, no positive bottles were detected in BacT/ALER system and only 1/35 (2.9%) in the VersaTREK system, but the BacT/ALERT system showed a higher detection rate than the other two systems, accounting for 20/35 (57.1%) (*P*=0.018). To ensure the activity of the antibiotics used in the experiments, antibiotic solutions and bacterial suspensions were co-incubated in VersaTREK aerobic bottles and anaerobic bottles. The results showed no bacterial growth in any of the culture bottles (data are not shown).

**Table 1:** Recovery in Aerobic (BacT/Alert FA Plus, BD BACTEC Aerobic/F Plus and VersaTREK Aerobic) or Anaerobic (BacT/Alert FN Plus, BD BACTEC Lytic/10 Anaerobic and VersaTREK Anaerobic) bottles containing antibiotics by microorganism.

Strains	AE			AN		
	BacT/ALERT	BD BACTEC	Versa TREK	BacT/ALERT	BD BACTEC	Versa TREK
*E. coli*	5/10 (50.0)	5/10 (50.0)	3/10 (30.0)	10/10 (100.0)	0/10 (0.0)	0/10 (0.0)
*P. aeruginosa*	5/10 (50.0)	5/10 (50.0)	5/10 (50.0)	0/10 (0.0)	0/10 (0.0)	0/10 (0.0)
*S. aureus*	5/5 (100.0)	5/5 (100.0)	0/5 (0.0)	5/5 (100.0)	0/5 (0.0)	0/5 (0.0)
*S. pneumoniae*	10/10 (100.0)	10/10 (100.0)	4/10 (40.0)	5/10 (50.0)	0/10 (0.0)	1/10 (10.0)
Total	25/35 (71.4)	25/35 (71.4)	12/35 (34.3)	20/35 (57.1)	0/35 (0.0)	1/35 (2.9)
*P*-value	0.047			0.018		

AE, aerobic; AN, anaerobic.

As shown in **Table 2**, the recovery rates of cefoperazone/sulbactam and penicillin in BacT/ALERT media were both 100%. The recovery rate of cefoperazone/sulbactam was 10/20 (50%) and 6/20 (30%) for BACTEC and VersaTREK media, respectively. The results showed significant differences in the recovery rates of cefoperazone/sulbactam in aerobic and anaerobic bottles in all three blood culture systems (*P <*0.05). While the recovery of penicillin was 5/10 (50%) and 2/10 (20%) in BACTEC and VersaTREK system, similarly, the recovery of penicillin in the three blood culture systems was significantly different (*P*<0.05). Vancomycin was highly recovered in the BacT/ALERT medium 15/20 (75%), whereas it was recovered in BACTEC and VersaTREK medium at 10/20 (50%) and 3/20 (15%), respectively. Imipenem showed the lowest recovery in the three blood culture systems, with a recovery of 5/20 (25%) in the BacT/ALERT system, but it was not recovered in either the BACTEC or VersaTREK systems. The above results revealed that the recovery of antibiotics by the BacT/ALERT system was higher than that of the BACTEC and VersaTREK systems.

**Table 2:** Recovery in aerobic (BacT/Alert FA Plus, BD BACTEC aerobic/F plus and VersaTREK aerobic) or anaerobic (BacT/alert FN plus, BD BACTEC lytic/10 anaerobic and VersaTREK anaerobic) bottles containing antibiotics by microorganism.

Strains	Antimicrobials	Vacuum environment	BacT/ALERT	BD BACTEC	Versa TREK	*P*
*E. coli*	Imipenem	AE	0/5	0/5	0/5	NA
		AN	5/5	0/5	0/5	0.001
	Cefoperazone/sulbactam	AE	5/5	5/5	3/5	0.006
		AN	5/5	0/5	0/5	0.001
*P. aeruginosa*	Imipenem	AE	0/5	0/5	0/5	NA
		AN	0/5	0/5	0/5	NA
	Cefoperazone/sulbactam	AE	5/5	5/5	3/5	0.006
		AN	5/5	0/5	0/5	0.001
*S. aureus*	Vancomycin	AE	5/5	5/5	0/5	0.001
		AN	5/5	0/5	0/5	0.001
*S. pneumoniae*	Vancomycin	AE	5/5	5/5	2/5	0.024
		AN	0/5	0/5	1/5	NA
	Penicillin	AE	5/5	5/5	2/5	0.024
		AN	5/5	0/5	0/5	0.001
Total		AE	25/35	25/35	12/35	0.001

AE, aerobic; AN, anaerobic; NA, not applicable.

**Table 3** showed that a total of five microorganism–antibiotic combinations were detected in the three aerobic culture systems. Among them, the TTD of both BacT/ALERT systems were shorter than the other two systems with three (60%) microorganism-antibiotic combinations. The TTD of BacT/Alert FA Plus bottles (24.4 h) was 5.1 h shorter than that of the BD BACTEC Aerobic/F Plus bottles (29.5 h), 34.1 h shorter than that of the VersaTREK Aerobic (58.5 h) (*P*=0.363). For the three anaerobic culture systems, five microorganism-antibiotic combinations were also detected, whereas four of these combinations were detected by the BacT/ALERT system; the TTD was<30 h for all four combinations. The TTD of the BacT/Alert FN Plus bottles was 17.6 h, 4.5 h longer than the TTD of the VersaTREK Anaerobic bottles (13.1 h) (*P*=0.033), whereas only one microorganism-antibiotic combination was detected in the VersaTREK Anaerobic bottles. BacT/Alert FN Plus bottles detected four combinations, while none of the BD BACTEC Lytic/10 Anaerobic bottles were detected.

**Table 3:** Average TTD of each microorganism-antibiotic combinations in aerobic and anaerobic bottles.

Strains	Antimicrobials	Average TTD (h)							
		AE				AN			
		BacT/ALERT	BD BACTEC	Versa TREK	*P*	BacT/ALERT	BD BACTEC	Versa TREK	*P*
*E. coli*	Imipenem	> 120	> 120	> 120	0.363	10.7	> 120	> 120	0.033
	Cefoperazone/sulbactam	11.9	42.8	65.2		12.6	> 120	> 120	
*P. aeruginosa*	Imipenem	> 120	> 120	> 120		> 120	> 120	> 120	
	Cefoperazone/sulbactam	14.5	13.5	28.1		> 120	> 120	> 120	
*S. aureus*	Vancomycin	17.8	27.9	> 120		18.8	> 120	> 120	
*S. pneumoniae*	Vancomycin	61	41.5	25.6		> 120	> 120	13.1	
	Penicillin	16.6	21.8	115		28.4	> 120	> 120	

AE, aerobic; AN, anaerobic; TTD, time to detection.

**Table 4** summarizes the differences in TTD for microorganism-antibiotic combinations and antibiotic-free controls for blood culture bottles (△TTD). For gram-negative bacteria, ΔTTD more than 8 h was observed for *E. coli*-cefoperazone/sulbactam in BD BACTEC systems, and *E. coli*-cefoperazone/sulbactam, *P. aeruginosa*-cefoperazone/sulbactam in VersaTREK systems. For gram-positive bacteria, ΔTTD more than 8 h was observed for *S. pneumoniae*-vancomycin and *S. pneumoniae*-penicillin in BacT/ALERT systems, *S. aureus*-vancomycin, *S. pneumoniae*-vancomycin, *S. pneumoniae*-penicillin in BD BACTEC systems, and *S. pneumoniae*-vancomycin, *S. pneumoniae*-penicillin in VersaTREK systems. In addition, we found that *P. aeruginosa* was detected in VersaTREK Anaerobic bottles, probably due to the poor anaerobic environment of the bottles.

**Table 4:** Average TTD of each antimicrobial-free control in aerobic and anaerobic bottles.

Strains	Antimicrobials	Average TTD (h)											
		BacT/ALERT				BD BACTEC				Versa TREK			
		Antimicrobial-free control		△TTD (h)		Antimicrobial-free control		△TTD (h)		Antimicrobial-free control		△TTD (h)	
		AE	AN	AE	AN	AE	AN	AE	AN	AE	AN	AE	AN
*E. coli*	Imipenem	10.3	10.6	NA	0.1	12.3	9.0	NA	NA	14.3	12.4	NA	NA
	Cefoperazone/sulbactam	11.3	11.6	0.6	1.0	11.7	10.2	31.1	NA	11.7	13.7	53.5	NA
*P. aeruginosa*	Imipenem	12.6	Neg	NA	NA	13.1	Neg	NA	NA	14.3	21.8	NA	NA
	Cefoperazone/sulbactam	12.2	Neg	2.3	NA	13.0	Neg	0.5	NA	11.9	18.7	16.2	NA
*S. aureus*	Vancomycin	13.1	15.3	4.7	3.5	15.4	10.6	12.5	NA	9.2	13.9	NA	NA
*S. pneumoniae*	Vancomycin	11.3	12.2	49.7	NA	9.1	11.0	32.4	NA	11.1	13.0	14.5	0.1
	Penicillin	13.4	14.1	3.2	14.3	11.9	15.5	9.9	NA	14.4	17.6	100.6	NA

AE, aerobic; AN, anaerobic; TTD, time to detection; △TTD, the difference in TTD between microorganism - antibiotic combinations blood culture and the antimicrobial - free control blood culture. NA, not applicable.

**Figure 1:**
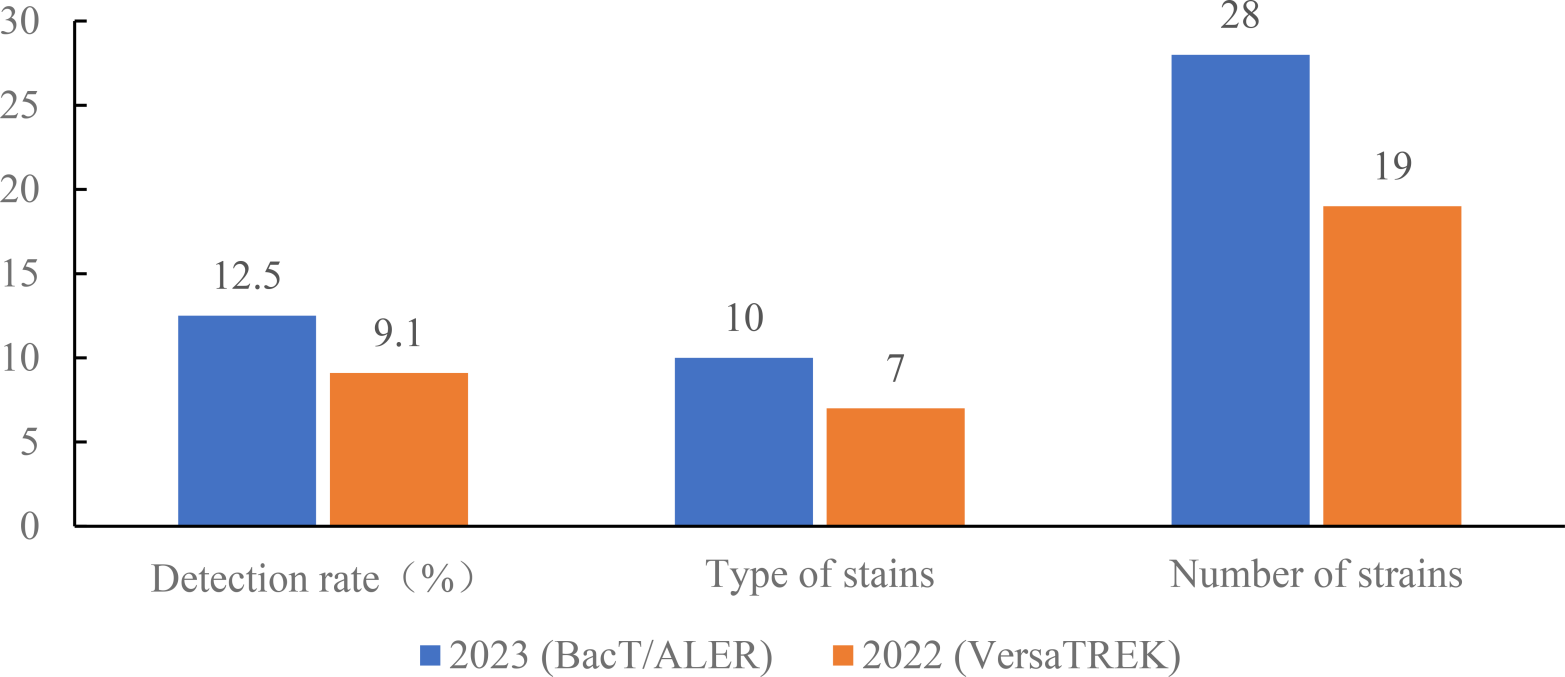
Comparison of the detection rate, species and number of bacteria detected in the comprehensive ICU using BacT/ALERT and VersaTREK medium, respectively.

**Figure 2:**
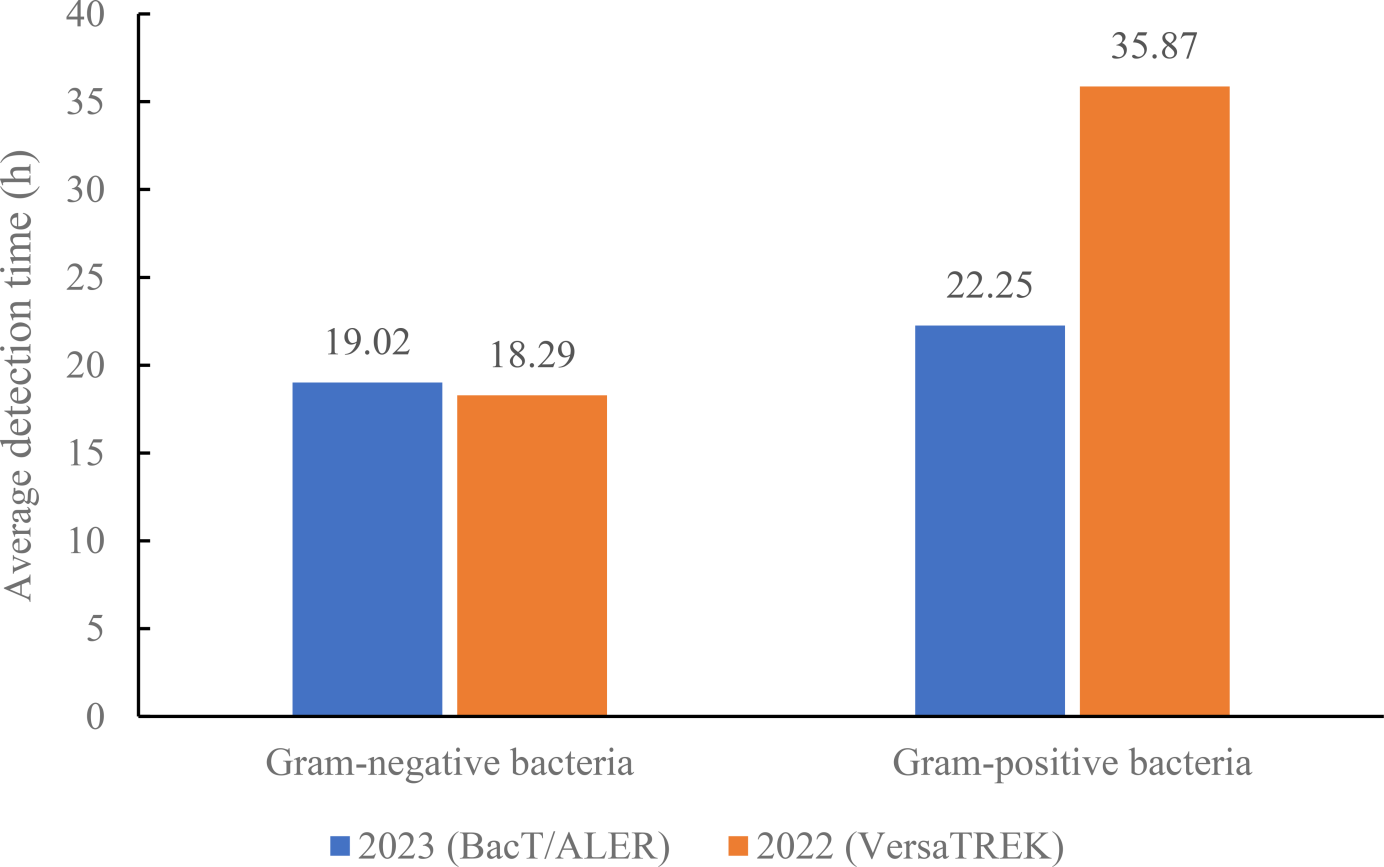
Comparison of average detection times of gram-negative and gram-positive bacteria in the comprehensive ICU using BacT/ALERT and VersaTREK medium.

The above studies demonstrate that the BacT/ALERT system blood culture bottles showed better adsorption and neutralization capabilities against antibiotics, resulting in shorter detection times for bacterial positivity in the microorganism-antibiotic combinations. Retrospective analysis of the data showed that the number of blood cultures sent for testing of the VersaTREK system was higher in the comprehensive ICU than in other departments. Therefore, we selected the blood culture bottles of the BacT/ALERT system used for one month in the comprehensive ICU in June 2023. Then, we compared blood culture results using the VersaTREK system in June 2022 with those using the BacT/ALERT system in June 2023 in the comprehensive ICU. In total, 151 pairs of blood culture bottles from the comprehensive ICU were tested in June 2023, as shown in **Figure 1**, and 18 pairs of positive blood cultures were detected. The detection rate was 11.92%, including 10 species of strains, totaling 28 strains. A total of 110 pairs of blood culture bottles were tested in June 2022 and 10 pairs of positive blood cultures were detected, a detection rate of 9.10%, including 8 species of strains, 19 strains in total. For gram-negative bacteria in the detected strains, the mean TTD for the BacT/ALERT system was 19.02 h, while the average TTD for the VersaTREK system was 18.29 h (**Figure 2**). The average TTD for gram-positive bacteria was 22.25 h and 35.87 h in the BacT/ALERT and VersaTREK systems, respectively (**Figure 2**). The above results showed that the detection rate of the BacT/ALER system was greater than the VersaTREK system, and the TTD of the BacT/ALER system was shorter than for the VersaTREK system for gram-positive bacteria. It is noteworthy that the difference in TTD between the two systems for gram-negative bacteria was not significantly different.

**Figure 3:**
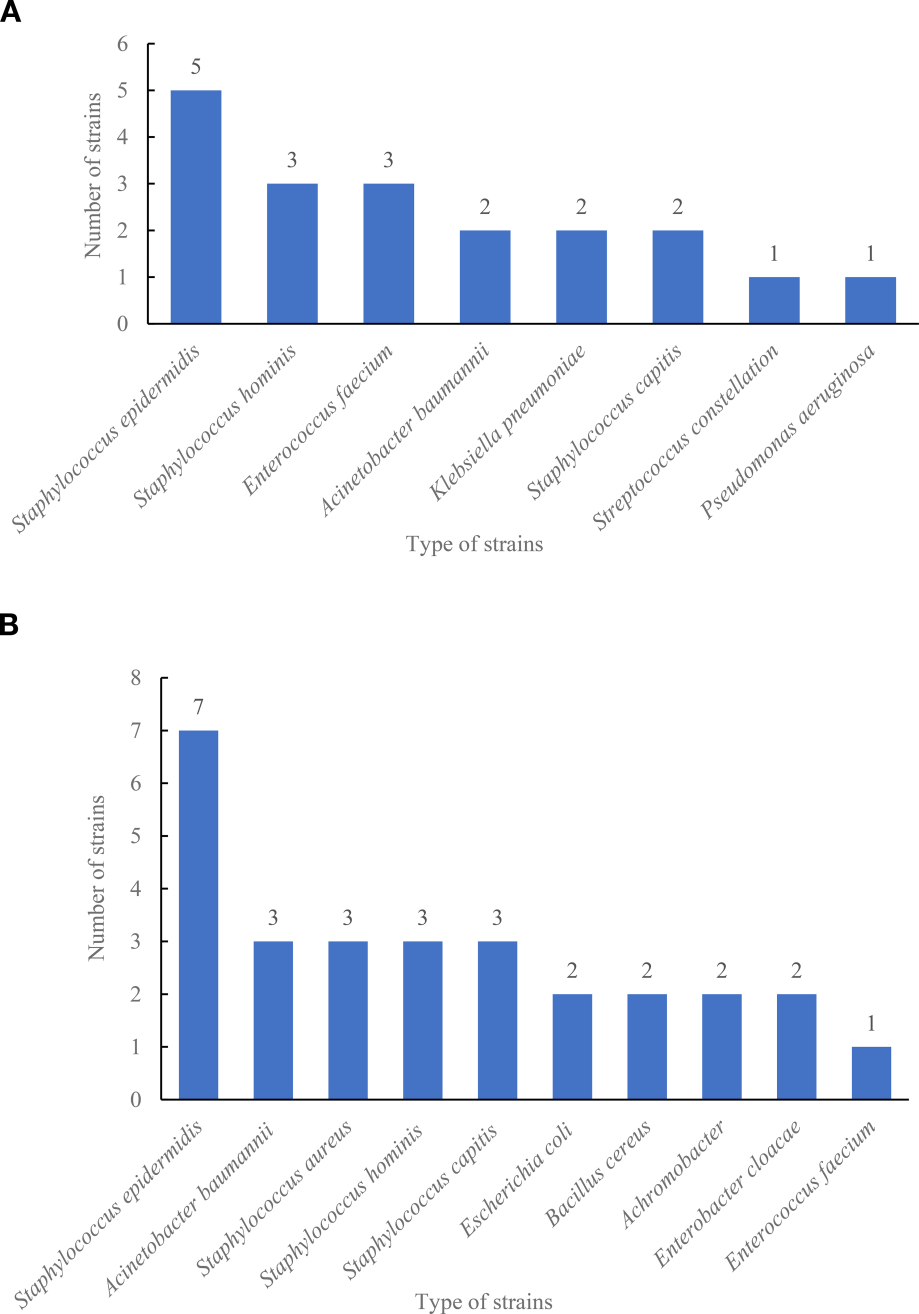
Type and number of strains detected in the comprehensive ICU using BacT/ALERT and VersaTREK medium. **(A)** Type and number of strains detected by VersaTREK medium. **(B)** Type and number of strains detected by BacT/ALERT medium.

We further compared the strains detected by the two systems. As shown in **Figure 3A**, the highest types of strains detected by the VersaTREK system in 2022 were *Staphylococcus epidermidis* detected five times, followed by *Staphylococcus hominis* and *Enterococcus faecium*, both detected three times. The highest count of strains detected by BacT/ALER system in 2023 was *Staphylococcus epidermidis* on seven occasions, while *Acinetobacter baumannii*, *Staphylococcus aureus*, *Staphylococcus hominis* and *Staphylococcus capitis* were all three times (**Figure 3B**). The results showed that the BacT/ALER system detected more strain types and at a greater rate than the VersaTREK system.

Next, we counted the number of patients with positive blood cultures in the comprehensive ICU during June 2022 and June 2023, and retrospectively analyzed their cases (**Table 5**). The statistics revealed that in June 2022, among patients tested using the VersaTREK system, 10 patients were positive with blood cultures, including 5 patients died, and 3 of them died before the blood culture results were reported. In June 2023, using the VersaTREK system, 17 patients were positive with blood cultures, including 8 patients died, and 4 of them died before the blood culture results were reported. Due to the limited sample, it cannot be concluded that using the VersaTREK system for blood culture detection enables patients to receive immediate treatment and reduces mortality rates.

**Table 5:** Case information for comprehensive ICU patients with positive blood cultures in June 2022 and June 2023.

(A)					
Gender	Year	Admission cause	TTD (h)	Bacteria	Discharge outcome
Woman	68	Septicemia	32.64	*Pseudomonas aeruginosa*	Transfer
			9.3	*Klebsiella pneumoniae*	
			8.9	*Klebsiella pneumoniae*	
Woman	63	Tick bite	7.92	*Acinetobacter baumannii*	Transfer
Woman	73	Acute diffuse peritonitis	69.3	*Staphylococcus hominis*	Transfer
Woman	74	Pulmonary infection	27.12	*Staphylococcus epidermidis*	Improvement
Man	32	Traffic accident injury	21.12	*Staphylococcus epidermidis*	Improvement
Woman	45	Acute pancreatitis	32.16	*Staphylococcus capitis*	Death
			13.44	*Acinetobacter baumannii*	
Man	33	Acute pancreatitis	45.8	*Staphylococcus epidermidis*	Death
			27.3	*Enterococcus faecium*	
Woman*	63	Pesticide poisoning	69.6	*Streptococcus constellation*	Death
Woman*	71	Tick bite	21.6	*Staphylococcus hominis*	Death
Woman*	72	Heart failure	16	*Staphylococcus hominis*	Death
(B)					
Gender	Year	Admission cause	TTH (h)	Bacteria	Discharge outcome
Man	85	Stomachache	16.8	*Escherichia coli*	Transfer
Man	68	Infective endocarditis	10.08	*Staphylococcus aureus*	Transfer
Man	70	Septicemia	17.76	*Staphylococcus epidermidis*	Transfer
			36	*Staphylococcus epidermidis*	
Man	31	Septic shock	10.8	*Bacillus cereus*	Transfer
Man	41	Traffic accident injury	18.3	*Staphylococcus capitis*	Transfer
Woman	58	Traffic accident injury	60	*Staphylococcus hominis*	Transfer
Man	43	Acute pancreatitis	39	*Staphylococcus epidermidis*	Transfer
Woman	54	Traffic accident injury	28.08	*Achromobacter*	Improvement
Woman	45	Traffic accident injury	28.8	*Staphylococcus hominis*	Improvement
Man	83	Severe pneumonia	17	*Enterococcus faecium*	Death
Man	53	Severe pneumonia	36.72	*Staphylococcus capitis*	Death
Woman	59	Rheumatoid arthritis	4.08	*Acinetobacter baumannii*	Death
Woman	52	Dermatomyositis	6.96	*Enterobacter cloacae*	Death
Woman*	59	After coronary artery bypass grafting	13.44	*Acinetobacter baumannii*	Death
Man*	43	Drug overdose	31.44	*Staphylococcus epidermidis*	Death
Man*	63	Intracranial space-occupying lesions	24.24	*Staphylococcus epidermidis*	Death
Man*	69	Right pulmonary occupying lesions	18.48	*Staphylococcus aureus*	Death
			18.72	*Staphylococcus epidermidis*	

(A) Case information for comprehensive ICU patients with positive blood cultures in June 2022; (B) Case information for comprehensive ICU patients with positive blood cultures in June 2023. *means that the patient died before the results of the blood cultures showed up.

## 4 Discussion

Although new microbiological techniques continue to evolve, blood cultures remains the gold standard for the diagnosis of BSIs (Dargere et al., 2018). Currently, increasing drug resistance leads to a higher incidence of serious infections, and it has never been more important to provide timely and effective antimicrobial drug therapy. According to the guideline’s best practice statement, it is recommended that blood cultures be performed before initiating antimicrobial therapy in patients with suspected BSIs (Evans et al., 2021). In the present study, we compared the detection rates of three blood culture systems by simulating blood specimens from patients with BSIs, with the BacT/ALERT system having the highest detection rate. Patients in an ICU usually have more severe conditions, and in this study, through checking the cases, we found that empirical antibiotic treatment was given before the collection of blood cultures. Surveys worldwide have also shown low compliance rates for blood cultures collected before antibiotic administration (Phua et al., 2011; Scheer et al., 2019). Early antibiotic therapy may shorten the time of treatment, prevent infectious shock and contribute to the reduction in mortality of patients with BSIs (Ferrer et al., 2014; Whiles et al., 2017). However, it has been shown that when blood cultures were positive before antimicrobial therapy, the detection rate of blood cultures was reduced by 50% after empirically administered therapy (Cheng et al., 2019). Therefore, in the present study, we also simulated blood specimens from patients with BSIs after antibiotic treatment and compared the recovery rates of antibiotics produced by the three blood culture systems. The results unequivocally showed that the BacT/ALERT system had a higher detection rate as well as a shorter detection time for antibiotics relative to the VersaTREK and BACTEC systems. Antibodies, complement, phagocytes, lysozyme and anti-microbial factors contained in blood inhibit or kill pathogenic microorganisms and also affect the positive detection rate of blood cultures. In order to minimize the impact of blood components on the detection rate, the commercial blood culture bottles currently contain basic nutrients as well as other components, such as anticoagulants, which prevent phagocytosis, inhibit complement and lymphocyte activity, and reduce the activity of aminoglycosides and polymyxins. The addition of resin or activated charcoal adsorbs free antimicrobials from the bloodstream and promotes bacterial growth. Furthermore, the addition of hemolysin lyses the cells and facilitates the release of intracellular infected microorganisms, and also dilutes the concentration of antibiotics in the blood, increasing the positive detection rate. Of the three blood culture bottles used in the present study, BacT/ALERT medium and BACTEC medium contained resin and anticoagulant, and VersaTREK medium contained hemolysin. The results showed that the medium containing resin and anticoagulant produced better recovery of antibiotics than the medium containing hemolysin. It was clear that the BacT/ALERT system produced higher detection rates and detection times compared to the other two systems. In addition, **Figure 1** shows that the detection rate and the number of detected species of blood cultures collected from comprehensive ICU patients with BacT/ALERT medium were higher than those using VersaTREK medium. There was no significant difference in the detection time of gram-negative bacteria between the two media, whereas the detection time of gram-positive bacteria was significantly less in BacT/ALERT medium than in VersaTREK medium. Therefore, in cases where blood cultures are collected after antibiotic therapy, the selection of appropriate blood culture bottles is crucial in order to improve the positive detection rate and TTD. We certainly still recommend that blood cultures be collected prior to antibiotic therapy whenever possible, to facilitate the efficacy of antimicrobial therapy and the safe downgrading of therapeutic regimens. For patients in the ICU or those with severe manifestations of BSIs, we recommend immediate antimicrobial therapy after blood cultures are collected as soon as possible.

Blood culture contamination (BCC) is one of the most serious problems during the blood culture process, which poses certain risks to the patients, such as some unnecessary antimicrobial treatments and hospitalization costs (Bates et al., 1991; Souvenir et al., 1998; Lee et al., 2007; Ai et al., 2024). The contaminant is any microorganism that enters the culture medium during specimen collection or processing that is not pathogenic to the patient (Dargere et al., 2018). Based on many years of research, the microorganisms isolated from blood that are most likely to represent contamination include most of the coagulase-negative staphylococci (CoNS), most of the *Corynebacterium* spp., *Bacillus* spp. (excluding *Bacillus anthracis*), *Micrococcus* spp. and *Propionibacterium* spp (Weinstein et al., 1997; Pien et al., 2010; Doern et al., 2019; Tenderenda et al., 2022; Aiesh et al., 2023). **Figure 3** also shows that the number of CoNS, such as *Staphylococcus epidermidis* and *Staphylococcus hominis* detected by both the BacT/ALERT and VersaTREK blood culture systems was higher. Review of cases revealed that some patients were treated with targeted antimicrobial therapy for the CoNS that were detected. Multiple studies have found that patients started on antibiotic therapy due to BCC received longer-term treatment (Pliakos et al., 2018; Doern et al., 2019; Liaquat et al., 2022). This can lead to an increase in antibiotic exposure, which may cause the emergence of antibiotic resistance and destabilize the microflora in patients. Contamination may be due to improper sampling, failure to follow strict aseptic practices, overstaffing and overcrowding in the area where blood is collected, such as the emergency department, or difficulty in collecting blood from children and elderly patients (Garcia et al., 2015; McLeod, 2020; Aiesh et al., 2023). Thus, we recommend that skin disinfection be performed in strict accordance with the guidelines, with enhanced training of nurses to follow the principles of asepsis during blood culture collection. It should be noted that 70% isopropyl alcohol was applied to sterilize the top of the blood culture bottles before collecting blood cultures (Clinical practice guideline: prevention of blood culture contamination, 2018; Doern et al., 2019). They should be sent to the laboratory for testing immediately after collection. Moreover, blood cultures should be performed as early as possible for critically ill patients with suspected BSIs. As shown in **Table 5**, about 30% of the patients with positive blood cultures of patients in the contamination ICU from June 2022 to June 2023 in our study died before the results of the blood cultures were obtained, which may also be related to the underlying disease, condition and age of the patients.

There are several limitations to our study. It mainly included fewer bottles in the microbial-antibiotic combinations and experimental groups, a single clinical department for statistical data, and only one month’s worth of data analyzed for comparisons. Therefore, the research as a whole had a limited amount of data to more fully confirm that the BacT/ALERT system was the best in terms of detection rate and time.

## 5 Conclusions

Although there are some limitations to the present study, the BacT/ALERT system was clearly shown to have some advantages in terms of detection rates and times, and had excellent adsorption for most of the antibiotics investigated. For patients who had blood cultures collected after antibiotic therapy, the blood culture bottles with better adsorption or dilution of antibiotics should be selected as far as possible. Although the early administration of antibiotics to critically ill patients is beneficial, blood cultures should be collected prior to the administration of antibiotics in order to prevent the development of antibiotic resistance and increase the length of hospitalization of patients. In addition, doctor and nurse training should be strengthened, and the aseptic operational procedure should be strictly implemented to provide patients with optimal treatment plans.

## Data Availability

The original contributions presented in the study are included in the article/**Supplementary Material**. Further inquiries can be directed to the corresponding authors.
