# Supplementary material for: Comparative study of antibiotic adsorption capacity of three brands of blood culture bottles
Source: Front Cell Infect Microbiol. 2025 Oct 8;15:1571264. doi: 10.3389/fcimb.2025.1571264 (PMC12540367; doi:10.3389/fcimb.2025.1571264)
Supplement: Supplementary file 1 [file Table1.docx]

Supplementary

**Table S1.** Primary components of different blood culture bottles. (a) The component of BacT/ALERT FA Plus; (b) The component of BacT/ALERT FN Plus; (c) The component of BD BACTECTM Plus Aerobic/F Culture Vials; (d) The component of BD BACTECTM Lytic/10 Anaerobic/F Culture Vials; (e) The component of Versa TREK REDOX 1(Aerobic bottles); (f) The component of Versa TREK REDOX 2 (Anaerobic bottles)

**(A)**

| Component | Content |
| --- | --- |
| Complex medium | 30 mL |
| Polymer adsorption beads | ≥1.6g |
| Peptone/biological extract mixture | ≥1.85% w/v |
| Anticoagulant | ≥0.083% w/v |
| Vitamins and amino acids | ≥0.00145% w/v |
| Carbon source | ≥0.45% w/v |
| Trace elements | ≥0.0005% w/v |

The culture bottles contain a vacuum environment mixed with N₂, O₂, and CO₂.

**(B)**

| Component | Content |
| --- | --- |
| Complex medium | 40 mL |
| Polymer adsorption beads | ≥1.6g |
| Peptone/biological extract mixture | ≥1.85% w/v |
| Anticoagulant | ≥0.083% w/v |
| Vitamins and amino acids | ≥0.00145% w/v |
| Carbon source | ≥0.45% w/v |
| Trace elements | ≥0.0005% w/v |

The culture bottles contain a vacuum environment mixed with N₂ and CO₂.

**(C)**

| Component | Content |
| --- | --- |
| Distilled water | 25 mL |
| Soy-casein-hydrolyzed broth | 2.75% w/v |
| Yeast extract | 0.25% w/v |
| Glucose | 0.06% w/v |
| Sucrose | 0.084% w/v |
| Hemin | 0.0005% w/v |
| Vitamin K3 | 0.00005% w/v |
| Pyridoxal hydrochloride (Vitamin B6) | 0.001% w/v |
| Sodium polyoxyethylene anisate sulfonate (SPS) | 0.05% w/v |
| Non-ionic adsorption resin | 16.0% w/v |
| Cation exchange resin | 1.0% w/v |

All BACTEC media are filled with CO₂.

**(D)**

| Component | Content |
| --- | --- |
| Distilled water | 40 mL |
| Soy-casein-hydrolyzed broth | 2.75% w/v |
| Yeast extract | 0.2% w/v |
| Animal tissue decomposition | 0.05% w/v |
| Glucose | 0.2% w/v |
| Hemin | 0.0005% w/v |
| Vitamin K3 | 0.00005% w/v |
| Sodium citrate | 0.02% w/v |
| Mercaptan | 0.1% w/v |
| Sodium pyruvate | 0.1% w/v |
| Saponin | 0.26% w/v |
| Demister | 0.01% w/v |
| Sodium anisole sulfonate | 0.035% w/v |

Pre-reduced in anaerobic medium with added CO₂ and N₂

**(E)**

| Component | Content |
| --- | --- |
| Treated water | 80 ml |
| Soybean-casein peptide | 2.1%w/v |
| Sodium chloride | 0.5%w/v |
| Yeast extract | 0.1%w/v |
| Glucose | 0.25%w/v |
| Divalent salt A | 0.009%w/v |
| Additive O | 0.33%w/v |
| Sodium anisole sulfonate | 0.0125%w/v |

**(F)**

| Component | Content |
| --- | --- |
| Treated water | 80 ml |
| Prion protein peptone | 1.5%w/v |
| Yeast extract | 0.5%w/v |
| Sodium chloride | 0.23%w/v |
| Glucose | 0.5%w/v |
| 10% Polysorbate 80 | 0.075%w/v |
| Additive AN | 0.8%w/v |
| Trisodium citrate | 0.07%w/v |
| Saponin | 0.045%w/v |
| Hemin | 0.0005%w/v |
| Cysteine | 0.05%w/v |
| Vitamin K | 0.0001%w/v |
| Resazurin | 0.0001%w/v |
